# Fhit–Fdxr interaction in the mitochondria: modulation of reactive oxygen species generation and apoptosis in cancer cells

**DOI:** 10.1038/s41419-019-1414-7

**Published:** 2019-02-15

**Authors:** Teresa Druck, Douglas G. Cheung, Dongju Park, Francesco Trapasso, Flavia Pichiorri, Marco Gaspari, Tiziana Palumbo, Rami I. Aqeilan, Eugenio Gaudio, Hiroshi Okumura, Rodolfo Iuliano, Cinzia Raso, Kari Green, Kay Huebner, Carlo M. Croce

**Affiliations:** 1 Department of Cancer Biology and Genetics, The Ohio State University Comprehensive Cancer Center, Columbus, OH 43210 USA; 2 Dipartimento di Medicina Sperimentale e Clinica, University “Magna Græcia” of Catanzaro, Catanzaro, 88100 Italy; 3 Department of Hematologic Malignancies Translational Science, Beckman Research Institute, City of Hope, Duarte, CA USA; 4 Dipartimento di Farmacologia Sperimentale Preclinica e Clinica, University of Catania, Catania, 95123 Italy; 5 Lautenberg Center for Immunology and Cancer Research, Faculty of Medicine, Hebrew University of Jerusalem, Jerusalem, Israel; 6 Università della Svizzera italiana, Institute of Oncology Research, Bellinzona, Switzerland; 7 Digestive Surgery, Breast and Thyroid Surgery, Graduate School of Medical Sciences, Kagoshima University, Sakuragaoka, Kagoshima, Japan; 8 Systems Biology Ireland, University College Dublin, Belfield, Dublin 4, Dublin, Ireland; 9 Department of Chemistry, Mass Spectrometry Research and Education Center, University of Florida, 126 Sisler Hall, Gainesville, FL 32611-7200 USA

## Abstract

Fhit protein is lost in cancers of most, perhaps all, cancer types; when restored, it can induce apoptosis and suppress tumorigenicity, as shown in vitro and in mouse tumor models in vivo. Following protein cross-linking and proteomics analyses, we characterized a Fhit protein complex involved in triggering Fhit-mediated apoptosis. The complex includes the heat-shock chaperonin pair, HSP60/10, which is likely involved in importing Fhit into the mitochondria, where it interacts with ferredoxin reductase, responsible for transferring electrons from NADPH to cytochrome P450 via ferredoxin, in electron transport chain complex III. Overexpression of Fhit protein in Fhit-deficient cancer cells modulates the production of intracellular reactive oxygen species, causing increased ROS, following peroxide treatment, with subsequent increased apoptosis of lung cancer cells under oxidative stress conditions; conversely, Fhit-negative cells escape ROS overproduction and ROS-induced apoptosis, likely carrying oxidative damage. Thus, characterization of Fhit-interacting proteins has identified direct effectors of a Fhit-mediated apoptotic signal pathway that is lost in many cancers. This is of translational interest considering the very recent emphasis in a number of high-profile publications, concerning the role of oxidative phosphorylation in the treatment of human cancers, and especially cancer stem cells that rely upon oxidative phosphorylation for survival. Additionally, we have shown that cells from a Fhit-deficient lung cancer cell line, are sensitive to killing by exposure to atovaquone, thought to act as a selective oxidative phosphorylation inhibitor by targeting the CoQ10 dependence of the mitochondrial complex III, while the Fhit-expressing sister clone is resistant to this treatment.

## Introduction

The ~2-MB FHIT genomic locus straddles an active common fragile site at chromosome band 3p14.2^1,2^. Partially due to this genomic fragility, Fhit mRNA and/or protein expression is lost or reduced in large fractions of almost all types of human tumors, due to allelic loss, genomic rearrangement, promoter hypermethylation, or combinations thereof^3–5^. Fhit knockout mice show significantly increased susceptibility to cancer development^6,7^, and FHIT gene therapy prevents or reduces tumor burdens in carcinogen-exposed Fhit-deficient mice^8,9^. Fhit restoration by stable transfection in cancer cells has little effect on cell growth in vitro, unless cells are exposed to stress, including the stress of the nude mouse environment in vivo;^10^ viral-mediated Fhit restoration, a process that simultaneously supplies stress and Fhit expression, suppresses tumorigenesis in vivo and triggers apoptosis of many types of malignant cells in vitro^11–14^, including lung cancer cells. In lung hyperplastic lesions, DNA damage checkpoint genes are already activated, in parallel with, or following, DNA alteration at FRA3B within FHIT; in the hyperplastic lesions, selection for mutations in checkpoint proteins can then lead to neoplastic progression^15,16^. Evidence of the loss of FHIT alleles occurs in normal-appearing bronchial epithelial cells of smokers, prior to pathologic changes or alterations in the expression of oncogenes or other suppressor genes^17–19^. Similarly, normal-appearing breast epithelial cells of mammalian ducts adjacent to cancer or even far from an invasive cancer, often show loss of Fhit protein^20^. Fhit expression is downregulated by exposure to DNA-damaging agents^21^ and plays a role in response to such agents^22,23^, with Fhit-deficient cells escaping apoptosis and accumulating mutations. To identify proteins that interact with Fhit to affect downstream apoptotic pathways, we cross-linked proteins within cells, after induced or viral-mediated Fhit overexpression in lung cancer cells, or endogenous expression in colon cancer cells, and characterized proteins associated with Fhit, and pathways affected by them.

## Results

### Isolation of a Fhit protein complex

A549 lung cancer-derived cells, expressing low-level endogenous Fhit, and susceptible to apoptosis on exogenous Fhit expression^11^, were infected with AdFHIT or AdFHIT-His6^24^ and treated with dithiobis(succinimidyl propionate (DSP)), a cross-linker that crosses membranes and fixes proteins in complex in vivo. Cells were lysed and proteins isolated with nickel beads avid for the His6 epitope tag. Purified proteins were treated with dithiothreitol (DTT) to cleave DSP and dissociate the complex, and digested by trypsin; protein constituents were identified by liquid-chromatography tandem mass spectrometry (LC-MS/MS) (Table 1 and Supplemental material) and six proteins were identified, all with mitochondrial localization: HSP60 and 10, ferredoxin reductase (Fdxr), malate dehydrogenase (Mdh), electron-transfer flavoprotein (Etfb), and mitochondrial aldehyde dehydrogenase 2 (Aldh2); HSP60 and HSP10 are also distributed in the cytosol^25^. Since HSP60/10 complex acts as a chaperonin, we thought that interaction with this heat-shock protein complex might chaperone Fhit to the mitochondria, where Fdxr is important in electron transport. We thus confirmed the interaction of Fhit protein with these three candidate interactors.

**Table 1:** Candidate Fhit protein partners isolated through mass spectrometry

Protein	Accession no.	M_r_ kDa	Function/category	Subcellular localization	No. of identical peptides	Peptide sequences	Protein Mascot score	Sequence coverage
HSP60	NP_002147	60	60-kDa heat-shock protein	Cytosol/mitochondria	6	VGEVIVTK LSDGVAVLK IGIEIIKR VTDALNATR TVIIEQSWGSPK VGGTSDVEVNEKK	239	10%
Malate dehydrogenase (Mdh)	NP_005909	33	Catalyzes the reversible oxidation of malate to oxaloacetate	Mitochondrial matrix	8	ANTFVAELK IQEAGTEVVK VNVPVIGGHAGK IFGVTTLDIVR FVFSLVDAMNGK GCDVVVIPAGVPR AGAGSATLSMAYAGAR GYLGPEQLPDCLK	193	28%
Electron- transfer flavoprotein (Etfb)	NP_001976	28	Specific electron acceptor for mitochondrial dehydrogenases	Mitochondrial matrix	3	EIDGGLETLR VETTEDLVAK LSVISVEDPPQR	96	12%
HSP10	AAC96332	10	10-kDa heat-shock protein	Cytosol/mitochondria	3	GGEIQPVSVK VLQATVVAVGSGSK VVLDDKDYFLFR	92	34%
Mitochondrial aldehyde dehydrogenase 2 (Adh2)	NP_000681	55	Second enzyme of the major oxidative pathway of alcohol metabolism	Mitochondrial matrix	2	LADLIER LGPALATGNVVVMK	75	4%
Ferredoxin reductase (Fdxr)	P22570	54	First electron- transfer protein in all the mitochondrial p450 systems	Mitochondrial matrix	1	FGVAPDHPEVK	47	2%

Proteins selectively captured in the A549 Ad FHIT-H6-infected cells sample. Amino acid sequence of identified peptides, Mascot scores, and protein sequence coverage are listed

### Fhit subcellular localization

**Fig. 1:**
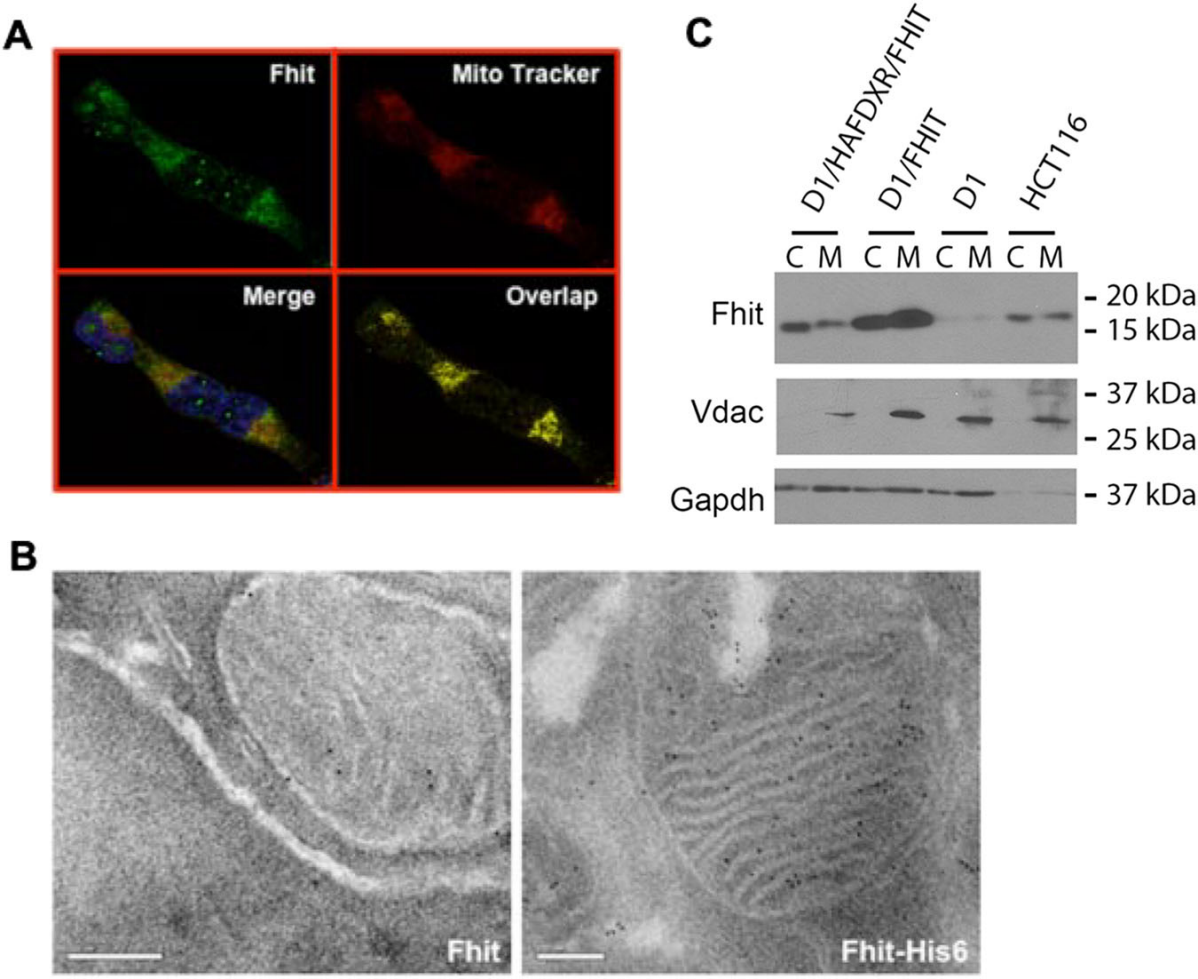
Subcellular localization of Fhit protein in the cytosol and mitochondria. **a** Immunofluorescence microscopy was performed with anti-Fhit serum on H1299 cells (D1) treated with PonA for 8 h; Fhit staining was detected using fluorescein isothiocyanate- (green) conjugated anti-rabbit immunoglobulin (IgG); MitoTracker Red staining, which identifies mitochondria, shows partial colocalization with Fhit. The yellow color on the fourth panel (lower right) shows the co-localization points. **b** Immuno-electron microscopy of A549 Ad*FHIT* (left) or Ad*FHIT-*His6*-*infected cells (right) performed with a Penta-His antibody shows Fhit mitochondrial localization (right); A549 cells infected with Ad*FHIT* served as a control and show only a few scattered grains (left). **c** Immunoblot analyses of cytosolic and mitochondrial protein fractions from H1299D1 and HCT116 cells. Vdac is a marker of the mitochondria

Since candidate Fhit interactors are mitochondrial proteins, we determined if Fhit, which lacks a mitochondrial localization signal, localized in these organelles, as reported previously^26^. Fhit-negative H1299 lung cancer cells carrying an inducible FHIT cDNA (D1 cells; refs. ^27^) were treated with the inducer, Ponasterone A (Pon A), for 48 h and indirect immunofluorescence detection of Fhit subcellular location was assessed using anti-Fhit serum and MitoTracker red 580, a mitochondria marker; Fhit fluorescent signal (green staining, Fig. 1a) was cytoplasmic and partly co-localized (yellow staining, Fig. 1a, lower right) with MitoTracker Red dye, indicating that exogenous Fhit localized to the mitochondria and cytosol. Anti-Fhit specificity was confirmed by the absence of green fluorescence in Fhit-negative H1299 clone E1 cells (not shown). To confirm mitochondrial localization, A549 lung cancer cells infected with AdFHIT-His6 or AdFHIT at multiplicity of infection (MOI) 20, were examined by immuno-electron microscopy 48 h later, by anti-penta-His staining; FhitHis6-transduced cells demonstrated significant numbers of gold particles in the mitochondria (Fig. 1b, right panel), while AdFHIT-transduced cells showed sparse reactivity (Fig. 1b, left panel). To assess Fhit submitochondrial localization, the mitochondria were purified from H1299D1 (inducible Fhit expression) and HCT116 (endogenous Fhit) cells (Fig. 1c). Fhit protein was detected in both the cytosolic and mitochondrial fractions.

### Fhit interacts with HSP60, HSP10, and Fdxr

**Fig. 2:**
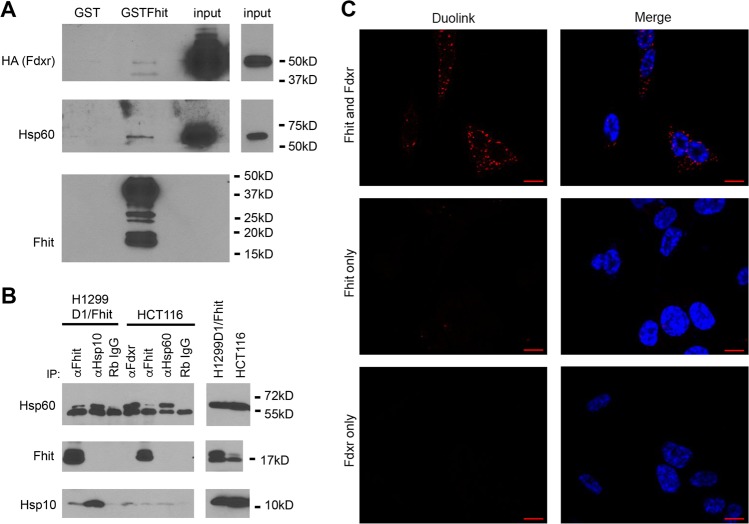
Fhit interacts with Fdxr, HSP10, and HSP60. **a** GST pull-down experiment using a protein lysate from H1299D1 cells transfected with HA-FDXR which was incubated with GST and GST-Fhit protein bound to glutathione agarose resin, and protein complexes eluted and separated on acrylamide gel for detection with antisera against HA, HSP60, and Fhit. **b** Lysates were prepared from DSP-cross-linked H1299D1 cells (Pon A induced for Fhit expression) or from HCT116 cells (expressing endogenous Fhit) and used in IP experiments with the indicated antisera. Complexes were separated on acrylamide gel and proteins were detected with HSP60, Fhit, or HSP10 antisera. **c** The association of Fhit and Fdxr in HCT116 cells using the Duolink in situ proximity ligation assay (PLA). PLA signals are shown in red and nuclei in blue. PLA signals for Fhit/Fdxr are mostly confined in the cytoplasm. Negative controls with the Fdxr or Fhit antibody alone show no PLA signal. Scale bar represents 10 μm

Among candidate interactor proteins, we focused on HSP60 and HSP10 chaperonins, and on Fdxr, a mitochondrial respiratory chain protein known to be transcriptionally activated by p53 and involved in responses to therapeutic drugs^28^. To validate interactions, we performed GST pulldown with lysates from p53-null^29^ H1299D1 cells transfected with HA-FDXR, and immunoprecipitation (IP) with lysates from PonA-treated, Fhit-induced H1299D1 cells and HCT116 cells that express endogenous Fhit. HSP60 and Fdxr were detected after pulldown with GST-Fhit (Fig. 2a). IP with anti-Fhit co-precipitated HSP10 and HSP60 in DSP-treated H1299D1/Fhit and HCT116 cells (Fig. 2b). Fdxr–Fhit interaction was also confirmed in HCT116 cells using the proximal ligation assay Duolink. This method uses binding of individual primary antibodies to the putative interacting proteins. Under a microscope, we observed punctate fluorescent spots, corresponding to antigens in closer proximity, only when the secondary antibodies were concomitantly recognizing the anti-Fhit and Fdxr antibodies; this effect was not observed when only one antigen was targeted (Fig. 2c)^30^.

### Fhit expression induces the generation of reactive oxygen species

**Fig. 3:**
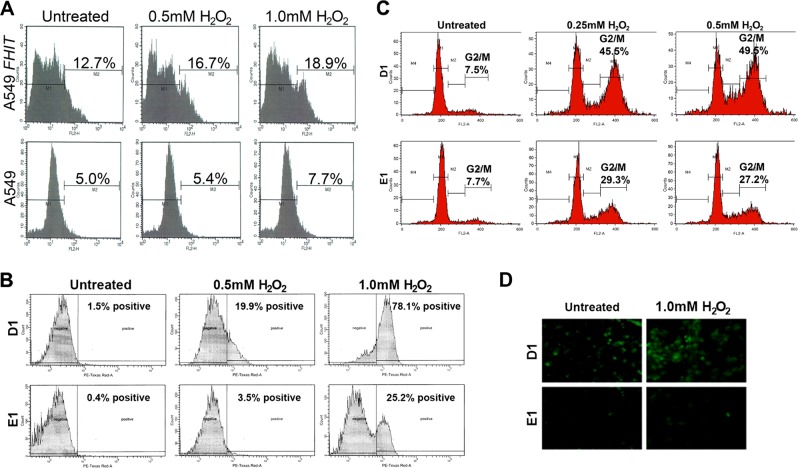
Fhit expression induces intracellular ROS generation after treatment of cells with peroxide. **a** FACS analysis for ROS assessment in A549 cells 48 h after transfection with *FHIT* plasmid, with or without 5 h of H_2_O_2_ treatment. Empty vector transfected cells served as a control. Intracellular superoxide was determined according to fluorescence of ethidium as a result of oxidation of hydroethidine by ·O_2_. M2 refers to the fraction of ROS-positive cells. **b** FACS analysis for ROS assessment by the fluorescence produced from the oxidation of hydroethidine in D1 and E1 cells; 48 h after PonA treatment, cells were treated for 5 h with 0.5 and 1.0 mM H_2_O_2_ and oxidative stress was measured; % positive refers to the fraction of fluorescent cells, indicating ROS. **c** FACS analysis of D1 and E1 cell cycle kinetics at 48 h after oxidative stress treatment. Cells were treated with PonA for 48 h and then with increasing concentrations of H_2_O_2_ (0.25, 0.5 mM) for 4 h. Analysis was at 48 h after H_2_O_2_ treatment. All experiments were performed twice in triplicate. **d** Increased green fluorescent DCF signal in H1299 Fhit-expressing cells (D1) under stress conditions. Cells were incubated with 2′,7′-dichlorodihydrofluorescein diacetate (H_2_DCFDA), a ROS indicator that can be oxidized in the presence of ROS to the highly green fluorescent dye dichlorofluorescein (DCF), at 48 h after Fhit induction and after 5 h of H_2_O_2_ treatment of E1 and D1 cells (×40 magnification)

Fdxr, a 54-kDa flavoprotein, is located on the matrix side of the inner mitochondrial membrane, and is responsible for transferring electrons from NADPH, via the single-electron shuttle ferredoxin–cytochrome P450, to substrates^31^. Under substrate-limiting conditions, electrons leak from this shuttling system and generate reactive oxygen species (ROS)^32^. Fdxr mediates p53-dependent, 5-fluorouracil (FU)-induced apoptosis in colorectal cancer cells, through generation of ROS^33,34^, potent intracellular oxidants, and regulators of apoptosis^35^. The discovery of the Fhit–Fdxr interaction prompted us to determine if ROS production could be involved in Fhit-mediated apoptosis. Overexpression of Fdxr increases the sensitivity of tumor cells to apoptosis following H_2_O_2_ treatment, through ROS production^33,34^. We examined ROS production in A549 cells, with or without H_2_O_2_ treatment, after transient transfection with Fhit expression plasmid. Intracellular superoxide (O_2_^–^) was assessed by measuring ethidium fluorescence, formed as a result of oxidation of hydroethydine by superoxide. Intracellular superoxide was measured 5 h after stimulation with increasing concentrations of H_2_O_2_. ROS generation was ~3 times higher (16.7 vs. 5.4% at 0.5 mM H_2_O_2_ and 18.9 vs. 7.7% at 1.0 mM H_2_O_2_) in Fhit-transfected cells (Fig. 3a).

A similar experiment was performed with p53-null, Fhit-negative lung cancer-derived H1299 D1 and E1 clones carrying PonA-inducible FHIT and empty vector expression plasmids, respectively; the cells were treated with 5 µM PonA and at 48 h treated with 0.5 and 1.0 mM H_2_O_2_; the percentage of ROS-positive cells was higher in Fhit-positive D1 cells than in E1 control cells (20% vs. 3.5% at 0.5 mM H_2_O_2_, and 78% vs. 25% at 1.0 mM H_2_O_2_, respectively) (Fig. 3b).

To further examine the generation of ROS after Fhit reconstitution during oxidative stress, dichlorofluorescein-diacetate (DCFH-DA) was used to measure the redox state of Fhit-overexpressing cells. Peroxidases, cytochrome C, and Fe^2+^ can oxidize DCFH-DA to fluorescent 2′-7′-dichlorofluorescein (DCF) in the presence of H_2_O_2_;^36^ thus, DCF indicates H_2_O_2_ levels and peroxidase activity. Increased DCF fluorescence was detected in D1 cells compared with E1 cells under stress conditions (Fig. 3d). To determine if H_2_O_2_ treatment with or without Fhit could affect cell viability or cell cycle kinetics, we performed flow cytometry (Fig. 3c); when Fhit was present under stress conditions, there was a consistent increase of G2/M arrest at 48 h after 0.25 and 0.5 mM H_2_O_2_ treatment, 45.5% and 49.5%, respectively, compared with 29.3 and 27.2% of E1 cells under the same conditions. These results suggest that expression of Fhit could also enhance oxidative stress-mediated apoptosis.

### Effect of the drug atovaquone on Fhit-positive and Fhit-negative cells

Atovaquone, an FDA-approved antimalarial drug, mainly used for the treatment of pneumocystis pneumonia or toxoplasmosis, is a hydroxy-1,4-naphthoquinone analog of ubiquinone, known as Co-enzyme Q10 (CoQ10). It acts as a potent, selective oxidative phosphorylation (OXPHOS) inhibitor through targeting of CoQ10 dependence of the mitochondrial electron transport complex (ETC) III^37^. Fiorillo et al.^37^ have recently shown that atovaquone has anticancer activity that is directed against cancer stem cells. Additionally, this study showed that atovaquone treatment of breast cancer cells inhibited oxygen consumption and induced aerobic glycolysis as well as oxidative stress. Since another very recent report^38^ showed that an inhibitor of OXPHOS, IACS-010759, a small-molecule inhibitor of ETC complex I, inhibited proliferation and induced apoptosis of brain cancer and AML cancer cells, which are dependent on OXPHOS, we were encouraged to try treatment of our Fhit-positive and Fhit-negative lung cancer cells with atovaquone, the ETC III inhibitor, with the idea that Fhit-negative cells may be dependent on OXPHOS for energy metabolism and survival. Recent evidence has revealed that cells harboring an unstable genome can be sensitized with reagents that enhance ROS production to induce cell death^39^.

**Fig. 4:**
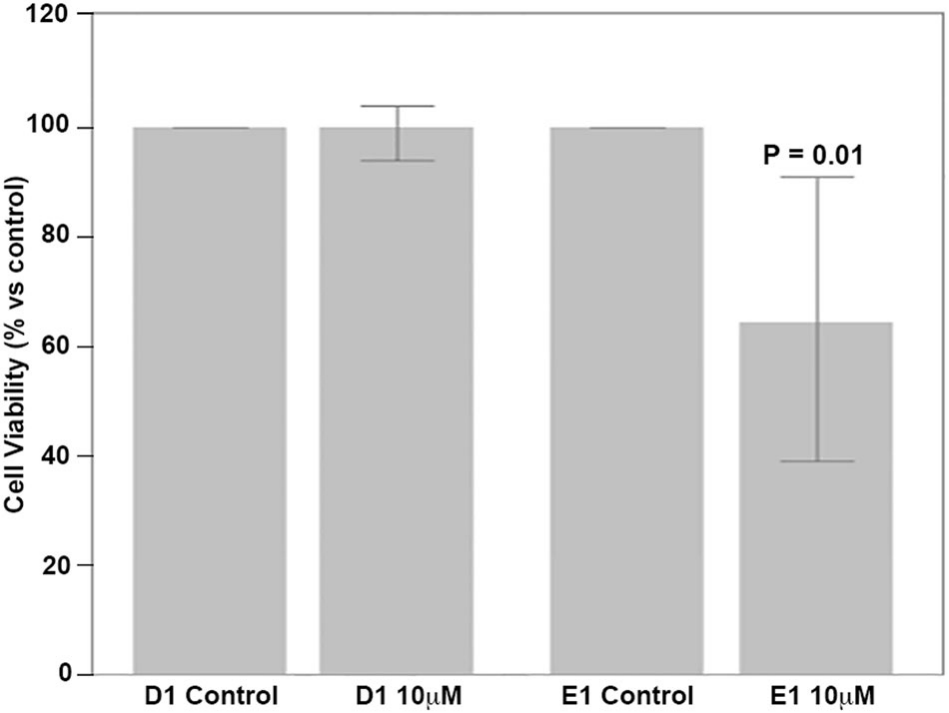
Cell viability of Fhit-negative H1299 cells is decreased after atovaquone treatment. H1299 E1 and D1 cells were treated with atovaquone (10 μM) for 3 days. Cell viability was assessed by trypan blue staining. Data from three independent experiments. Note that the percentage of cell viability of atovaquone-exposed cells is normalized to the control DMSO-treated cells

Thus, we used our H1299 sister clones, D1 and E1 (carrying an inducible Fhit plasmid and the inducible empty vector, respectively^27^) to test this idea. The cells were first induced with 5 μM Pon A for 24 h to activate the inducible plasmid promoters, and 30 h later, the cells were treated with vehicle (DMSO) or vehicle plus atovaquone, as described^37^, and cell viability was assessed on day 3 following atovaquone treatment. As illustrated in Fig. 4, the atovaquone-treated Fhit-deficient E1 lung cancer cells showed significantly reduced viability, while the Fhit-positive cells were unaffected by atovaquone (see Fig. 1c for Fhit expression in these cells after Pon A treatment). These findings suggest that OXPHOS inhibitors could selectively target Fhit-negative tumor cells.

## Discussion

Earlier searches for Fhit-interacting proteins pointed to several candidate proteins, none of which we could confirm as interactors (unpublished data) by co-IP experiments, including Ubc9, α-tubulin, and Mdm2^40–42^. To readdress the question of Fhit protein interactors, we used adenovirus-transduced Fhit-His6 for Fhit complex purification after cross-linking, and Fhit-linked proteins, HSP60, HSP10, and Fdxr, were identified; the subcellular location of these proteins suggested that the mitochondria might be a focus of Fhit activity. HSP “stress proteins” as molecular chaperones perform functions, such as protein translocation, folding, and assembly^43^. The finding that Fhit interacts with HSP60/HSP10, suggests that the HSP complex may be important for Fhit stability, and possibly for its correct folding to import it into the mitochondria, prior to activation of an apoptotic pathway.

Targeted disruption of the FDXR gene in HCT116 colon cancer cells showed that it was essential for viability; reduction of the gene copy number resulted in decreased sensitivity to 5-FU-induced apoptosis^33^ and FDXR is a target gene of the p53 family^34^. Overexpression of Fdxr-sensitized colon cancer cells to H_2_O_2_, 5-FU, and doxorubicin-induced cell death, indicates that Fdxr contributes to p53-mediated apoptosis through generation of oxidative stress in the mitochondria. Thus, activated p53 induces apoptosis in response to cellular stresses in part through ROS, and simultaneously p53 increases transcription of the FDXR gene, which in turn enhances p53 function by increasing ROS-induced apoptosis^33,34^.

We have shown the presence of Fhit in the mitochondrial fraction. In H1299 cells, missing both Fhit and p53, Fdxr overexpression increases the sensitivity to ROS-induced cell death and H1299 cells expressing inducible Fhit or p53, are sensitive to ROS-induced cell death; cancer cells missing Fhit, p53, or both would lack ways to increase Fdxr expression, would be less sensitive to oxidative damage, and would survive.

Discovery of the mitochondrial function of Fhit in apoptosis, through interaction with Fdxr, extends functional parallels of tumor suppressors, Fhit and p53, lost sequentially in most cancers and involved in response to DNA damage, with p53 acting as a transcriptional and Fhit a posttranscriptional Fdxr regulator. Membrane-bound Fdxr accepts two electrons from NADPH, yielding NADP+ ; these electrons are passed to the iron–sulfur cluster of ferredoxin (Fdx), which donates the electrons to the heme prosthetic group of mitochondrial cytochrome p450 (CYP), which uses protons and molecular O_2_ to hydroxylate its target substrate, yielding the final hydroxylated product. The electron donor, Fdx, is a 14-kDa mitochondrial matrix protein containing a Fe–S cluster^44^.

**Fig. 5:**
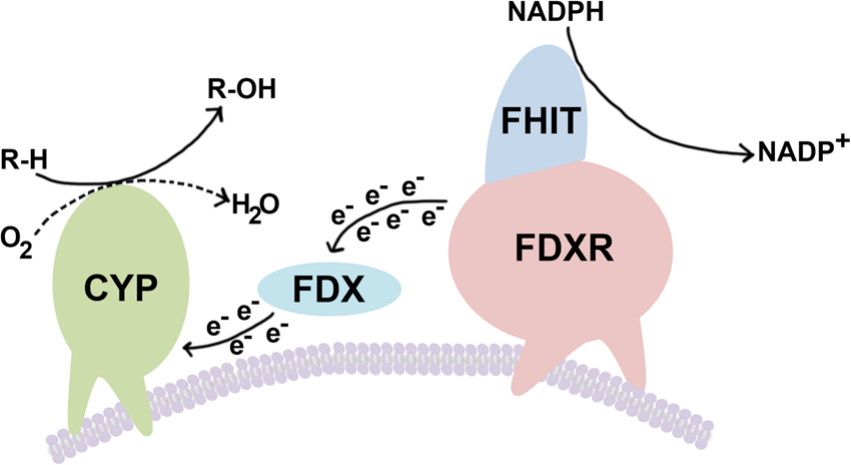
Mitochondrial cytochrome P450 (cyp) ETC. The model is modified from Midzak & Papadopoulos^44^. Membrane-bound ferredoxin reductase (Fdxr) accepts two electrons from NADPH, yielding NADP + ; these electrons are passed to the iron–sulfur cluster of ferredoxin (FDX), which donates the electrons to the heme prosthetic group of mitochondrial CYP, which uses protons and molecular O_2_ to hydroxylate its target substrate, yielding the final hydroxylated product, as described by Midzak & Papadopoulos^44^. The electron donor, FDX, is a 14-kDa mitochondrial matrix protein containing a Fe–S cluster. The mechanism of Fhit action on the Fdx–Fdxr interaction is unknown but perhaps Fhit interrupts this interaction and thus blocks the passing of electrons to Fdx, allowing leakage of electrons and contribution to ROS production and apoptosis, under specific conditions

Figure 5 depicts a model of the Fdxr–Fhit interaction, as a modulator of the Fdxr–Fdx interaction, and thus a modulator of the leakage of electrons from ETC III, which would contribute to ROS production and apoptosis in normal Fhit-positive cells, a modulation that would be absent in Fhit-deficient cells. Delineation of direct downstream effectors of the Fhit suppressor pathway will lead to further mechanistic studies of Fhit function that may influence preventive and therapeutic strategies for the many Fhit-deficient cancers of many types. The finding that ROS modulation is involved in Fhit-mediated apoptosis emphasizes the importance of Fhit loss as a negative prognostic factor in various clinical settings; for example, assessment of Fhit status in preneoplastic or neoplastic conditions may be predictive of the responses to antioxidant treatments.

There is a need for additional cellular targets for cancer prevention and treatment. Fhit expression is reduced or lost early in neoplasia development, leading to loss of a key ETC complex III OXPHOS interaction that participates in the production of protons and molecular O_2_ through the electron donor ferredoxin.

It has very recently been observed that “drugging mitochondrial oxidative phosphorylation might be relevant for cancer treatment” as reported by Molina et al.^38^. The authors noted that “this area has been unexplored, owing to an incomplete understanding of tumor contexts in which OXPHOS is essential”. This report characterized an inhibitor of complex I of the mitochondrial ETC, IACS-010759, and stated that treatment of cancer cells with it inhibited proliferation and induced apoptosis in brain cancer and acute AML models that rely on OXPHOS. Brain cancer and AML tumor growth was potently inhibited in vivo following IACS-010759 treatment and the authors noted that IACS-010759 is under evaluation in phase 1 clinical trials in relapsed/refractory AML and solid tumors. Thus, it is feasible that such Complex I inhibitors may prove useful, with further exploration, in cancer treatment. The authors concluded that: “Through development and characterization of IACS-010759”, they have provided evidence that there are tumor contexts that are dependent on OXPHOS for survival. In these tumor cells, “OXPHOS disruption created an environment of energy and macromolecule depletion that led to cell cycle arrest, apoptosis”.

Fhit protein is a modulator of ROS and thus of apoptotic responses to ROS^45,46^. Because Fhit expression is lost in most cancers^3,4^, including nearly all AMLs^5^, most cancers have lost this important ROS modulator and an important apoptotic signal pathway, allowing inappropriate survival and growth of cancers. Does this suggest that the absence of Fhit expression in AMLs could have been contributing to the OXPHOS dependency of the AML and brain cancers examined^38^, and contributing to the responses of these cancers to the Complex I inhibitor examined in this report? We do know that the H460 lung cancer cell line used in the Molina et al.^38^ experiments is completely negative for Fhit expression^10^. Studies of Fhit-deficient cancers for dependency on OXPHOS for survival, as described^38^, will identify Fhit-deficient cancer cell lines and cancers that can be examined for stratification by OXPHOS dependency and for responses to inhibitors of the mitochondrial electron transport chain complexes.

## Materials and methods

### Generation of recombinant adenovirus

The recombinant adenovirus carrying the wild-type FHIT cDNA (AdFHIT) was prepared as previously described^12^. A His-tagged FHIT cDNA was generated by PCR with the following oligonucleotides: 5′-ACgTggATCCCTgTgAggACATgTCgTTCAgATTTggC-3′(forward) and 5′-TTgTggATCCTTATCAgTgATggTgATggTgATgCgATCCTCTCTgAAAgTAgACCCgCAg-3′. These primers were designed with a *Bam*HI restriction site for subcloning into the transfer vector pAdenoVator-CMV5-IRES-GFP. The AdFHIT-His6 was generated with the AdenoVator^TM^ kit (Qbiogene, Carlsbad, CA), following the manufacturer’s procedure. AdGFP, used as a control, was purchased from Qbiogene.

### Cells

H1299D1 and E1 lung cancer cell clones were maintained as previously described^47^. A549 lung cancer expressing a reduced level of endogenous Fhit protein, and HCT116 colon cancer cells, expressing normal Fhit expression level, were maintained in RPMI-1640 medium plus 10% FBS and penicillin/streptomycin (Sigma, St. Louis, MO). HCT116/*FDXR*+/–/– cells were obtained from Bert Vogelstein and retain only one copy of the FDXR gene vs. three copies in the parental HCT116 colon cancer cell line^33^.

### Western blot analysis

Immunoblot analyses were performed as described^14^, using monoclonal anti-penta-His (Qiagen, Venlo, Netherlands), rabbit polyclonal anti-Fhit^48^, rabbit polyclonal antisera against GFP (Santa Cruz Biotechnology, Dallas, TX), HSP60 (Abcam ab109660, Cambridge, UK), HSP10 (Abcam, ab108600), Vdac (Cell Signaling #46615, Danvers, MA), rat anti-HA (Roche, Switzerland), and mouse anti-GAPDH (Calbiochem CB1001, San Diego, CA).

### Sample digestion and LC-MS/MS analysis

Proteins isolated with Ni-NTA beads were precipitated with cold acetone and resuspended in 6 M urea buffered at pH 8 with 0.1 M Tris-HCl. Protein reduction and alkylation were achieved, respectively, by the addition of DTT (final concentration 10 mM, 1 h of incubation at 37 °C) and iodoacetamide (final concentration 25 mM, 1 h of incubation at 37 °C). After neutralizing excess iodoacetamide with DTT (additional 5 mM), urea concentration was lowered to 1.5 M by dilution with 1 mM CaCl_2_. Overnight digestion was carried out using 50 ng of TPCK-treated trypsin (Sigma). The total digestion solution volume was 100 μl.

Chromatography was performed on an Ultimate nano-LC system from Dionex (Sunnyvale, CA). Digest mixtures (30 µl) were directly injected onto a Pepmap C18 RP cartridge (0.3-mm ID × 5-mm length) and washed for 10 min with H_2_O/trifluoroacetic acid (TFA)/acetonitrile 97.9:0.1:2 (v/v/v) before the RP trap was switched online to a 75-µm × 150-mm Pepmap C18 nano-LC column. Gradient elution of peptides was achieved at 300 nl/min using a 45-min linear gradient going from 5% B to 50% B. Mobile phase A was H_2_O/acetonitrile/formic acid (FA)/TFA 97.9:2:0.08:0.02 (v/v/v/v); mobile phase B was H_2_O/acetonitrile/FA/TFA 4.9:95:0.08:0.02 (v/v/v/v).

MS detection was performed on an Applied Biosystems (Framingham, MA) QSTAR XL hybrid LC-MS/MS operating in positive ion mode, with nanoelectrospray potential at 1800 V, curtain gas at 15 units, and CAD gas at 3 units. Information-dependent acquisition was performed by selecting the two most abundant peaks for MS/MS analysis after a full TOF-MS scan from 400 to 1200 *m/z* lasting for 2 s. Both MS/MS analyses were performed in enhanced mode (2 s/scan).

MS/MS spectra were searched by interrogating the Swiss Protein database on the Mascot search engine (www.matrixscience.com). The following search parameters were used. MS tolerance: 50 ppm; MS/MS tolerance: 1 Da; methionine oxidized (variable modification); cysteine carbamidomethylated (fixed modification); enzyme: trypsin; maximum missed cleavages: 1. Protein lists obtained from, respectively, both A549 infected with Ad FHIT-His6 and control were compared, and proteins exclusively present in the A549-Ad FHIT-His6 list were kept for further validation. As a first validation procedure, LC-MS/MS raw data were inspected using selected ion chromatogram (SIC) displaying mode. By SIC comparison, it could be assessed for the exclusive presence of the peptides of interest, identified as belonging to the six candidate proteins under examination, in the Ad FHIT-His6 sample (data not shown). These findings were further validated by biochemical and functional assays. Additional LC-MS/MS experiments and analyses were performed in the Ohio State University shared protein core and interactions were confirmed by IP experiments (data not shown).

### Immunoprecipitation

Proteins were extracted in M-PER (Pierce) or RIPA buffer. Co-immunoprecipitation (Co-IP) experiments, with or without dithiobis(succinimidyl propionate) (DSP) (Thermo Scientific, Waltham, MA) were performed by incubating 1 mg of total proteins with HSP60 (Abcam, ab109660), HSP10 (Abcam, ab108600), Fhit^48^, and HA antisera conjugated with protein A/G ultralink resin (Thermo Scientific) overnight at 4 °C; after TBST washing, beads were boiled in 1× reducing sample buffer and proteins were separated on polyacrylamide gel, transferred to a membrane, and probed with specific antisera.

### GST pulldown

In total, 150 μg of GST or GST-Fhit protein was bound to glutathione agarose (50% slurry; Thermo Scientific) following the manufacturer’s instructions. Equal amounts of protein lysate were added to the columns and rotated at 4 °C overnight. The resin was washed five times in a 1:1 mixture of TBS (50 mM Tris-Cl, 150 mM NaCl) and RIPA (EMD Millipore, Burlington, MA) buffers and eluted in 1× RSB buffer (2% SDS, 60 mM Tris-Cl, 5% glycerol, and 3% β-mercaptoethanol) for western blotting.

### Subcellular localization of Fhit protein

Fhit was sublocalized in Pon A-induced, Fhit-expressing H1299 D1 cells by indirect immunofluorescence detection using anti-Fhit serum and by detection of FhitHis6 in A549 Ad*FHIT*-His6 infected cells in immuno-electron micrographs using anti-penta-His. See Supplementary information for details. In fractionation studies, mitochondria were isolated with the Mitochondria/Cytosol Fractionation kit (Thermo Scientific).

### Duolink in situ proximity ligation assay (PLA)

HCT116 cells were cultured on poly-L-lysine-coated glass coverslips for 24 h. Cells were fixed in 4% paraformaldehyde in PBS (pH 7.4) for 15 min at room temperature, and permeabilized in 0.5% Triton X-100 in PBS for 15 min. The PLA procedure followed the manufacturer’s recommended protocol (Sigma). Rabbit polyclonal anti-Fhit^48^ was used at 1:400 and mouse monoclonal anti-Fdxr at 1:40 (Santa Cruz, sc-374436). Cells were viewed and photographed with an Olympus spectral FV1000 confocal microscope with 100× objective lens and images were processed with the Fiji ImageJ program.

### Flow cytometry

PonA-induced H1299 D1 and E1 cells were treated with H_2_O_2_ and incubated for varying times, as indicated in the text and figures. Cells were collected, washed with PBS, and resuspended in cold 70% ethanol. For analysis, cells were spun down, washed in PBS, and suspended in 0.1 mg/ml propidium iodide/Triton X-100 staining solution (0.1% Triton X-100, 20 mg/mL, 0.2 mg/mL DNase-free RNase A) for 30 min at room temperature and analyzed by flow cytometry.

### Assessment of intracellular ROS

Intracellular superoxide was measured through ethidium fluorescence as a result of oxidation by hydroethidine (dihydroethidium-HE; Molecular Probes, Eugene, OR). A549 cells transiently expressing Fhit and H1299-inducible Fhit-expressing cells were treated with 0.5 and 1.0 mM H_2_O_2_ at 37 °C; 4 h later, hydroethidine (10 μM) was added to the cells and incubated for 15 min at 37 °C. Fluorescence was measured by flow cytometry. DCFH-DA (Molecular Probes) was used in D1 cells expressing induced Fhit, stressed with H_2_O_2_ (0.1–1.0 mM), treated with 10 μM DCFH-DA, and incubated for 1 h at 37 °C. DCF fluorescence was measured by flow cytometry on a FACScan flow cytometer and fluorescence microscopy.

### Cell viability

H1299 E1 and D1 cells were seeded at a density of 45,000 cells/well in a six-well plate. To induce Fhit in D1 cells, PonA was added to all cells and 30 h later, cells were treated with DMSO (vehicle) or atovaquone (10 μM). Cell viability was measured 3 days later by trypan blue staining and automated cell counting. The percentage of cell viability of atovaquone-exposed cells is normalized to the value of control DMSO-treated cells. Experiments were performed three times. *P*-value was calculated by Student’s *t* test.

## Supplementary information

Supplemental Methods and Data
