# Supplementary material for: Fhit–Fdxr interaction in the mitochondria: modulation of reactive oxygen species generation and apoptosis in cancer cells
Source: Cell Death Dis. 2019 Feb 15;10(3):147. doi: 10.1038/s41419-019-1414-7 (PMC6377664; doi:10.1038/s41419-019-1414-7)
Supplement: Supplementary file 1 — Supplemental Methods and Data [file 41419_2019_1414_MOESM1_ESM.docx]

**Supplemental Data**

##### Fhit interaction with ferredoxin reductase triggers generation of reactive oxygen species and apoptosis of cancer cells

**Materials and methods**

**Mitochondrial localization studies**

Confocal microscopy was used to assess Fhit protein distribution by immunofluorescence; H1299 D1 cells, with inducible FHIT cDNA, and E1 cells, with empty vector, were treated with PonA for 48 hr, and living cells were stained with Mitotracker Red 580 (M-22425, Molecular Probes, Eugene, OR) at a working concentration of 500 nM for 40 min under growth conditions. The cells were fixed and permeabilized by incubation in ice-cold acetone for 5 min and then washed in PBS. Cells were incubated for 1 hr with 5% BSA to block non-specific interactions and than incubated overnight with Fhit antiserum (Zymed, S. San Francisco, CA) at a working concentration of 1.6 µg/ml, washed with PBS and incubated with Alexa Fluor 488 donkey anti-rabbit IgG (Molecular Probes). The slides were mounted in mounting medium for fluorescence with DAPI (Vector, Burlingane, CA) and visualized. For immuno electron microscopy localization of Fhit, A549 cells infected with Ad*FHIT-*His6 or Ad*FHIT,* MOI 5, were fixed in 4% paraformaldehyde in PBS pH7.2 for 30 min at 4°C, washed 3 times with PBS, and remaining free aldehyde groups were reduced using a 30 min incubation in 0.05% sodium borohydride in PBS. Following a PBS wash, samples were blocked with 50 mM glycine in PBS for 30 min, washed twice in PBS, and dehydrated with 25 and 50% ethanol for 15 min each, followed by 3 changes of 70% ethanol for 15 min each. The samples were then infiltrated with 70% ethanol+LR White resin, hard grade (Electron Microscopy Sciences, Hatfield, PA) at 2:1 for 1 hr, 70% ethanol + LR White at 1:2 for 1 hr, 100% LR White for 1 hr and 100% LRW overnight at 4oC. The following day the cells received 2 more changes of 100% LR White and were polymerized in gelatin capsules at 58°C for 20-24 hr. 900 nm thin sections were cut using a Reichert UCT Ultramicrotome and a diamond knife and placed on nickel grids. The grids were floated section side down on drops of PBS for 5 min, 5% goat serum in PBS for 1 hr at room temperature (RT), and either Penta-His mouse monoclonal antibody at 20 mg/ml (Qiagen, Valencia, CA) diluted in PBS containing 0.1% BSA and 0.05% Tween-20 (BSA/Tw) or BSA/Tw alone overnight at 4oC in a humidified chamber. The following day the grids were washed 6x for 5 min each using PBS and then incubated with goat anti-mouse 10 nm colloidal gold conjugate (Ted Pella, Redding, CA) diluted 1:10 in BSA/Tw for 2 hrs at RT. The grids were washed 6X each for 5 min with PBS, rinsed with DI H2O and post-stained with 2.5% aqueous uranyl acetate for 3 min. Images were collected on a Tecnai 12 electron microscope equipped with a US1000 Gatan 2K digital camera.

### List of proteins selectively identified by Mascot in the Ad FHIT-His6 sample.

#### Individual peptide ions scores > 26 indicate identity or extensive homology (p<0.05). Protein scores are derived from ions scores as a non-probabilistic basis for ranking protein hits.

CH60_HUMAN **Mass:** 61187 **Score:** 239 **Queries matched:** 6 **emPAI:** 0.34

60 kDa heat shock protein, mitochondrial precursor - Homo sapiens (Human) Check to include this hit in error tolerant search

| **Query** | **Observed** | **Mr(expt)** | **Mr(calc)** | **ppm** | **Miss** | **Score** | **Expect** | **Rank** | **Peptide** |
| --- | --- | --- | --- | --- | --- | --- | --- | --- | --- |
| 25 | **422.7441** | **843.4737** | **843.5066** | **-39.02** | **0** | **74** | **1.5e-06** | **1** | **K.VGEVIVTK.D** |
| 37 | **451.2572** | **900.4999** | **900.5280** | **-31.22** | **0** | **26** | **0.096** | **1** | **K.LSDGVAVLK.V** |
| 48 | **471.2977** | **940.5808** | **940.6069** | **-27.81** | **1** | **64** | **5.1e-06** | **1** | **K.IGIEIIKR.T** |
| 56 | **480.7451** | **959.4756** | **959.5036** | **-29.21** | **0** | **81** | **3.2e-07** | **1** | **R.VTDALNATR.A** |
| 178 | **672.8447** | **1343.6749** | **1343.7085** | **-25.04** | **0** | **77** | **6e-07** | **1** | **R.TVIIEQSWGSPK.V** |
| 183 | **681.3275** | **1360.6404** | **1360.6834** | **-31.61** | **1** | **51** | **0.00024** | **1** | **K.VGGTSDVEVNEKK.D** |

MDHM_HUMAN **Mass:** 35965 **Score:** 193 **Queries matched:** 8 **emPAI:** 1.01 Malate dehydrogenase, mitochondrial precursor - Homo sapiens (Human)

Check to include this hit in error tolerant search

| **Query** | **Observed** | **Mr(expt)** | **Mr(calc)** | **ppm** | **Miss** | **Score** | **Expect** | **Rank** | | **Peptide** |  |
| --- | --- | --- | --- | --- | --- | --- | --- | --- | --- | --- | --- |
| 64 | **496.7585** | **991.5024** | **991.5338** | **-31.67** | **0** | **56** | **9.4e-05** | **1** | **R.ANTFVAELK.G** | | |
| 93 | **537.2764** | **1072.5383** | **1072.5764** | **-35.55** | **0** | **27** | **0.075** | **1** | **R.IQEAGTEVVK.A** | | |
| 124 | **574.3109** | **1146.6073** | **1146.6510** | **-38.06** | **0** | **38** | **0.0044** | **1** | **R.VNVPVIGGHAGK.T** | | |
| 156 | **617.3395** | **1232.6644** | **1232.7129** | **-39.34** | **0** | **52** | **0.00016** | **1** | **K.IFGVTTLDIVR.A** | | |
| 173 | **664.3146** | **1326.6147** | **1326.6642** | **-37.32** | **0** | **50** | **0.00021** | **1** | **R.FVFSLVDAMNGK.E** | | |
| 174 | **669.8421** | **1337.6696** | **1337.7126** | **-32.13** | **0** | **54** | **0.00012** | **1** | **K.GCDVVVIPAGVPR.K** | | |
| 207 | **727.8348** | **1453.6551** | **1453.6983** | **-29.72** | **0** | **69** | **2.2e-06** | **1** | **K.AGAGSATLSMAYAGAR.F** | | |
| 215 | **745.3628** | **1488.7110** | **1488.7283** | **-11.59** | **0** | **32** | **0.014** | **1** | **K.GYLGPEQLPDCLK.G** | | |

ETFB_HUMAN **Mass:** 28054 **Score:** 96 **Queries matched:** 3 **emPAI:** 0.46 Electron transfer flavoprotein subunit beta - Homo sapiens (Human)

Check to include this hit in error tolerant search

| **Query** | **Observed** | **Mr(expt)** | **Mr(calc)** | **ppm** | **Miss** | **Score** | **Expect** | **Rank** | **Peptide** |
| --- | --- | --- | --- | --- | --- | --- | --- | --- | --- |
| 104 | **551.7746** | **1101.5346** | **1101.5666** | **-29.06** | **0** | **63** | **1.3e-05** | **1** | **R.EIDGGLETLR.L** |
| 105 | **552.7736** | **1103.5326** | **1103.5710** | **-34.81** | **0** | **43** | **0.0016** | **1** | **K.VETTEDLVAK.L** |
| 175 | **670.3428** | **1338.6711** | **1338.7143** | **-32.32** | **0** | **44** | **0.001** | **1** | **K.LSVISVEDPPQR.T** |

CH10_HUMAN **Mass:** 10925 **Score:** 92 **Queries matched:** 5 **emPAI:** 1.56

10 kDa heat shock protein, mitochondrial - Homo sapiens (Human) Check to include this hit in error tolerant search

| **Query** | **Observed** | **Mr(expt)** | **Mr(calc)** | **ppm** | **Miss** | **Score** | **Expect** | **Rank** | **Peptide** |
| --- | --- | --- | --- | --- | --- | --- | --- | --- | --- |
| 71 | **507.2695** | **1012.5245** | **1012.5553** | **-30.46** | **0** | **38** | **0.0033** | **2** | **K.GGEIQPVSVK.V** |
| 77 | **518.2844** | **1034.5542** | **1034.5913** | **-35.81** | **1** | **6** | **6.8** | **9** | **R.KFLPLFDR.V** |
| 95 | **538.7867** | **1075.5588** | **1075.5914** | **-30.23** | **0** | **18** | **0.6** | **1** | **K.VLLPEYGGTK.V** |
| 170 | **658.3645** | **1314.7144** | **1314.7507** | **-27.65** | **0** | **68** | **4.1e-06** | **1** | **K.VLQATVVAVGSGSK.G** |
| 224 | **765.3766** | **1528.7387** | **1528.7926** | **-35.25** | **1** | **38** | **0.0041** | **1** | **K.VVLDDKDYFLFR.D** |

ALDH2_HUMAN **Mass:** 56859 **Score:** 75 **Queries matched:** 2 **emPAI:** 0.14 Aldehyde dehydrogenase, mitochondrial precursor - Homo sapiens (Human)

Check to include this hit in error tolerant search

| **Query** | **Observed** | **Mr(expt)** | **Mr(calc)** | **ppm** | **Miss** | **Score** | **Expect** | **Rank** | **Peptide** |
| --- | --- | --- | --- | --- | --- | --- | --- | --- | --- |
| 23 | **415.2294** | **828.4442** | **828.4705** | **-31.70** | **0** | **47** | **0.00069** | **1** | **R.LADLIER.D** |
| 186 | **685.3756** | **1368.7367** | **1368.7799** | **-31.53** | **0** | **55** | **7.9e-05** | **1** | **K.LGPALATGNVVVMK.V** |

ADRO_HUMAN **Mass:** 54259 **Score:** 47 **Queries matched:** 1 **emPAI:** 0.07 NADPH:adrenodoxin oxidoreductase, mitochondrial precursor - Homo sapiens (Human) Check to include this hit in error tolerant search

| **Query** | **Observed** | **Mr(expt)** | **Mr(calc)** | **ppm Miss Score** | **Expect Rank** | **Peptide** |
| --- | --- | --- | --- | --- | --- | --- |
| 144 | **598.2873** | **1194.5601** | **1194.6033** | **-36.17 0 47** | **0.00049 1** | **R.FGVAPDHPEVK.N** |

FHIT_HUMAN **Mass:** 16905 **Score:** 65 **Queries matched:** 1 **emPAI:** 0.23 Bis(5'-adenosyl)-triphosphatase - Homo sapiens (Human)

Check to include this hit in error tolerant search

| **Query** | **Observed** | **Mr(expt)** | **Mr(calc)** | **ppm Miss Score Expect Rank** | **Peptide** |
| --- | --- | --- | --- | --- | --- |
| 189 | **689.3055** | **1376.5964** | **1376.6241** | **-20.12 0 65 3.6e-06 1** | **R.SEEEMAAEAAALR.V** |

**MS/MS spectra of the six peptides used for SIC comparison are reported below.**

##### Peptide View

MS/MS Fragmentation of **TVIIEQSWGSPK**

Found in **CH60_HUMAN**, 60 kDa heat shock protein, mitochondrial precursor - Homo sapiens (Human)

Match to Query 178: 1343.674864 from(672.844708,2+)


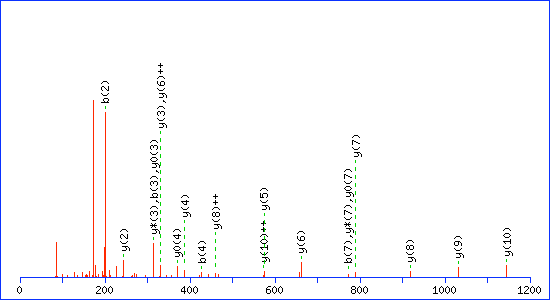


**Monoisotopic mass of neutral peptide Mr(calc):** 1343.7085

**Fixed modifications:** Carbamidomethyl (C)

**Ions Score:** 77 **Expect:** 6e-07

**Matches (Bold Red):** 21/118 fragment ions using 23 most intense peaks

| **#** | **b** | **b++** | **b*** | **b*++** | **b0** | **b0++** | **Seq.** | **y** | **y++** | **y*** | **y*++** | **y0** | **y0++** | **#** |
| --- | --- | --- | --- | --- | --- | --- | --- | --- | --- | --- | --- | --- | --- | --- |
| **1** | 102.0550 | 51.5311 |  |  | 84.0444 | 42.5258 | **T** |  |  |  |  |  |  | **12** |
| **2** | **201.1234** | 101.0653 |  |  | 183.1128 | 92.0600 | **V** | 1243.6681 | 622.3377 | 1226.6416 | 613.8244 | 1225.6575 | 613.3324 | **11** |
| **3** | **314.2074** | 157.6074 |  |  | 296.1969 | 148.6021 | **I** | **1144.5997** | **572.8035** | 1127.5732 | 564.2902 | 1126.5891 | 563.7982 | **10** |
| **4** | **427.2915** | 214.1494 |  |  | 409.2809 | 205.1441 | **I** | **1031.5156** | 516.2615 | 1014.4891 | 507.7482 | 1013.5051 | 507.2562 | **9** |
| **5** | 556.3341 | 278.6707 |  |  | 538.3235 | 269.6654 | **E** | **918.4316** | **459.7194** | 901.4050 | 451.2061 | 900.4210 | 450.7141 | **8** |
| **6** | 684.3927 | 342.7000 | 667.3661 | 334.1867 | 666.3821 | 333.6947 | **Q** | **789.3890** | 395.1981 | **772.3624** | 386.6849 | **771.3784** | 386.1928 | **7** |
| **7** | **771.4247** | 386.2160 | 754.3981 | 377.7027 | 753.4141 | 377.2107 | **S** | **661.3304** | **331.1688** | 644.3039 | 322.6556 | 643.3198 | 322.1636 | **6** |
| **8** | 957.5040 | 479.2556 | 940.4775 | 470.7424 | 939.4934 | 470.2504 | **W** | **574.2984** | 287.6528 | 557.2718 | 279.1396 | 556.2878 | 278.6475 | **5** |
| **9** | 1014.5255 | 507.7664 | 997.4989 | 499.2531 | 996.5149 | 498.7611 | **G** | **388.2191** | 194.6132 | 371.1925 | 186.0999 | **370.2085** | 185.6079 | **4** |
| **10** | 1101.5575 | 551.2824 | 1084.5310 | 542.7691 | 1083.5469 | 542.2771 | **S** | **331.1976** | 166.1024 | **314.1710** | 157.5892 | **313.1870** | 157.0972 | **3** |
| **11** | 1198.6103 | 599.8088 | 1181.5837 | 591.2955 | 1180.5997 | 590.8035 | **P** | **244.1656** | 122.5864 | 227.1390 | 114.0731 |  |  | **2** |
| **12** |  |  |  |  |  |  | **K** | 147.1128 | 74.0600 | 130.0863 | 65.5468 |  |  | **1** |

##### Peptide View

MS/MS Fragmentation of **LGPALATGNVVVMK**

Found in **ALDH2_HUMAN**, Aldehyde dehydrogenase, mitochondrial precursor - Homo sapiens (Human)

#### Match to Query 186: 1368.736748 from(685.375650,2+)


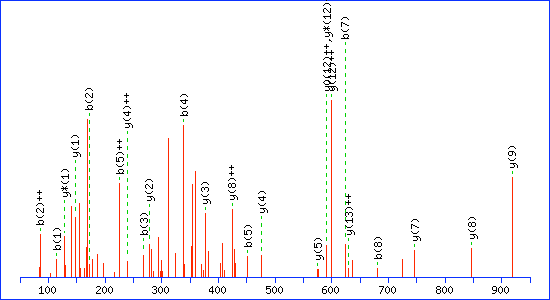


##### Monoisotopic mass of neutral peptide Mr(calc): 1368.7799

**Fixed modifications:** Carbamidomethyl (C)

**Ions Score:** 55 **Expect:** 7.9e-05

**Matches (Bold Red):** 24/114 fragment ions using 49 most intense peaks

| **#** | **b** | **b++** | **b*** | **b*++** | **b0** | **b0++** | **Seq.** | **y** | **y++** | **y*** | **y*++** | **y0** | **y0++** | **#** |
| --- | --- | --- | --- | --- | --- | --- | --- | --- | --- | --- | --- | --- | --- | --- |
| **1** | **114.0913** | 57.5493 |  |  |  |  | **L** |  |  |  |  |  |  | **14** |
| **2** | **171.1128** | **86.0600** |  |  |  |  | **G** | 1256.7031 | **628.8552** | 1239.6766 | 620.3419 | 1238.6926 | 619.8499 | **13** |
| **3** | **268.1656** | 134.5864 |  |  |  |  | **P** | 1199.6817 | **600.3445** | 1182.6551 | **591.8312** | 1181.6711 | **591.3392** | **12** |
| **4** | **339.2027** | 170.1050 |  |  |  |  | **A** | 1102.6289 | 551.8181 | 1085.6023 | 543.3048 | 1084.6183 | 542.8128 | **11** |
| **5** | **452.2867** | **226.6470** |  |  |  |  | **L** | 1031.5918 | 516.2995 | 1014.5652 | 507.7863 | 1013.5812 | 507.2942 | **10** |
| **6** | 523.3239 | 262.1656 |  |  |  |  | **A** | **918.5077** | 459.7575 | 901.4812 | 451.2442 | 900.4971 | 450.7522 | **9** |
| **7** | **624.3715** | 312.6894 |  |  | 606.3610 | 303.6841 | **T** | **847.4706** | **424.2389** | 830.4441 | 415.7257 | 829.4600 | 415.2337 | **8** |
| **8** | **681.3930** | 341.2001 |  |  | 663.3824 | 332.1949 | **G** | **746.4229** | 373.7151 | 729.3964 | 365.2018 |  |  | **7** |
| **9** | 795.4359 | 398.2216 | 778.4094 | 389.7083 | 777.4254 | 389.2163 | **N** | 689.4015 | 345.2044 | 672.3749 | 336.6911 |  |  | **6** |
| **10** | 894.5043 | 447.7558 | 877.4778 | 439.2425 | 876.4938 | 438.7505 | **V** | **575.3585** | 288.1829 | 558.3320 | 279.6696 |  |  | **5** |
| **11** | 993.5728 | 497.2900 | 976.5462 | 488.7767 | 975.5622 | 488.2847 | **V** | **476.2901** | **238.6487** | 459.2636 | 230.1354 |  |  | **4** |
| **12** | 1092.6412 | 546.8242 | 1075.6146 | 538.3109 | 1074.6306 | 537.8189 | **V** | **377.2217** | 189.1145 | 360.1952 | 180.6012 |  |  | **3** |
| **13** | 1223.6817 | 612.3445 | 1206.6551 | 603.8312 | 1205.6711 | 603.3392 | **M** | **278.1533** | 139.5803 | 261.1267 | 131.0670 |  |  | **2** |
| **14** |  |  |  |  |  |  | **K** | **147.1128** | 74.0600 | **130.0863** | 65.5468 |  |  | **1** |

MS/MS Fragmentation of **IFGVTTLDIVR**

Found in **MDHM_HUMAN**, Malate dehydrogenase, mitochondrial precursor - Homo sapiens (Human)

Match to Query 156: 1232.664392 from(617.339472,2+)


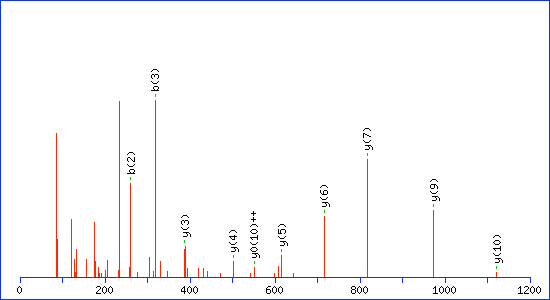


##### Monoisotopic mass of neutral peptide Mr(calc): 1232.7129

**Fixed modifications:** Carbamidomethyl (C)

**Ions Score:** 52 **Expect:** 0.00016

**Matches (Bold Red):** 10/86 fragment ions using 16 most intense peaks

| **#** | **b** | **b++** | **b0** | **b0++** | **Seq.** | **y** | **y++** | **y*** | **y*++** | **y0** | **y0++** | **#** |
| --- | --- | --- | --- | --- | --- | --- | --- | --- | --- | --- | --- | --- |
| **1** | 114.0913 | 57.5493 |  |  | **I** |  |  |  |  |  |  | **11** |
| **2** | **261.1598** | 131.0835 |  |  | **F** | **1120.6361** | 560.8217 | 1103.6095 | 552.3084 | 1102.6255 | **551.8164** | **10** |
| **3** | **318.1812** | 159.5942 |  |  | **G** | **973.5677** | 487.2875 | 956.5411 | 478.7742 | 955.5571 | 478.2822 | **9** |
| **4** | 417.2496 | 209.1285 |  |  | **V** | 916.5462 | 458.7767 | 899.5197 | 450.2635 | 898.5356 | 449.7715 | **8** |
| **5** | 518.2973 | 259.6523 | 500.2867 | 250.6470 | **T** | **817.4778** | 409.2425 | 800.4512 | 400.7293 | 799.4672 | 400.2373 | **7** |
| **6** | 619.3450 | 310.1761 | 601.3344 | 301.1709 | **T** | **716.4301** | 358.7187 | 699.4036 | 350.2054 | 698.4196 | 349.7134 | **6** |
| **7** | 732.4291 | 366.7182 | 714.4185 | 357.7129 | **L** | **615.3824** | 308.1949 | 598.3559 | 299.6816 | 597.3719 | 299.1896 | **5** |
| **8** | 847.4560 | 424.2316 | 829.4454 | 415.2264 | **D** | **502.2984** | 251.6528 | 485.2718 | 243.1395 | 484.2878 | 242.6475 | **4** |
| **9** | 960.5401 | 480.7737 | 942.5295 | 471.7684 | **I** | **387.2714** | 194.1394 | 370.2449 | 185.6261 |  |  | **3** |
| **10** | 1059.6085 | 530.3079 | 1041.5979 | 521.3026 | **V** | 274.1874 | 137.5973 | 257.1608 | 129.0840 |  |  | **2** |
| **11** |  |  |  |  | **R** | 175.1190 | 88.0631 | 158.0924 | 79.5498 |  |  | **1** |

MS/MS Fragmentation of **VLQATVVAVGSGSK**

Found in **CH10_HUMAN**, 10 kDa heat shock protein, mitochondrial - Homo sapiens (Human) Match to Query 170: 1314.714380 from(658.364466,2+)


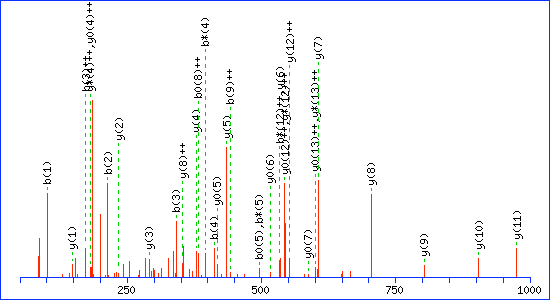


**Monoisotopic mass of neutral peptide Mr(calc):** 1314.7507

**Fixed modifications:** Carbamidomethyl (C)

**Ions Score:** 68 **Expect:** 4.1e-06

**Matches (Bold Red):** 33/142 fragment ions using 59 most intense peaks

| **#** | **b** | **b++** | **b*** | **b*++** | **b0** | **b0++** | **Seq.** | **y** | **y++** | **y*** | **y*++** | **y0** | **y0++** | **#** |
| --- | --- | --- | --- | --- | --- | --- | --- | --- | --- | --- | --- | --- | --- | --- |
| **1** | **100.0757** | 50.5415 |  |  |  |  | **V** |  |  |  |  |  |  | **14** |
| **2** | **213.1598** | 107.0835 |  |  |  |  | **L** | 1216.6896 | 608.8484 | 1199.6630 | **600.3352** | 1198.6790 | **599.8431** | **13** |
| **3** | **341.2183** | **171.1128** | 324.1918 | 162.5995 |  |  | **Q** | 1103.6055 | **552.3064** | 1086.5790 | **543.7931** | 1085.5949 | **543.3011** | **12** |
| **4** | **412.2554** | 206.6314 | **395.2289** | 198.1181 |  |  | **A** | **975.5469** | 488.2771 | 958.5204 | 479.7638 | 957.5364 | 479.2718 | **11** |
| **5** | 513.3031 | 257.1552 | **496.2766** | 248.6419 | **495.2926** | 248.1499 | **T** | **904.5098** | 452.7585 | 887.4833 | 444.2453 | 886.4993 | 443.7533 | **10** |
| **6** | 612.3715 | 306.6894 | 595.3450 | 298.1761 | 594.3610 | 297.6841 | **V** | **803.4621** | 402.2347 | 786.4356 | 393.7214 | 785.4516 | 393.2294 | **9** |
| **7** | 711.4400 | 356.2236 | 694.4134 | 347.7103 | 693.4294 | 347.2183 | **V** | **704.3937** | **352.7005** | 687.3672 | 344.1872 | 686.3832 | 343.6952 | **8** |
| **8** | 782.4771 | 391.7422 | 765.4505 | 383.2289 | 764.4665 | **382.7369** | **A** | **605.3253** | 303.1663 | 588.2988 | 294.6530 | **587.3148** | 294.1610 | **7** |
| **9** | 881.5455 | **441.2764** | 864.5189 | 432.7631 | 863.5349 | 432.2711 | **V** | **534.2882** | 267.6477 | 517.2617 | 259.1345 | **516.2776** | 258.6425 | **6** |
| **10** | 938.5669 | 469.7871 | 921.5404 | 461.2738 | 920.5564 | 460.7818 | **G** | **435.2198** | 218.1135 | 418.1932 | 209.6003 | **417.2092** | 209.1082 | **5** |
| **11** | 1025.5990 | 513.3031 | 1008.5724 | 504.7898 | 1007.5884 | 504.2978 | **S** | **378.1983** | 189.6028 | 361.1718 | **181.0895** | 360.1878 | **180.5975** | **4** |
| **12** | 1082.6204 | 541.8139 | 1065.5939 | **533.3006** | 1064.6099 | 532.8086 | **G** | **291.1663** | 146.0868 | 274.1397 | 137.5735 | 273.1557 | 137.0815 | **3** |
| **13** | 1169.6525 | 585.3299 | 1152.6259 | 576.8166 | 1151.6419 | 576.3246 | **S** | **234.1448** | 117.5761 | 217.1183 | 109.0628 | 216.1343 | 108.5708 | **2** |
| **14** |  |  |  |  |  |  | **K** | **147.1128** | 74.0600 | 130.0863 | 65.5468 |  |  | **1** |

MS/MS Fragmentation of **EIDGGLETLR**

Found in **ETFB_HUMAN**, Electron transfer flavoprotein subunit beta - Homo sapiens (Human) Match to Query 104: 1101.534580 from(551.774566,2+)


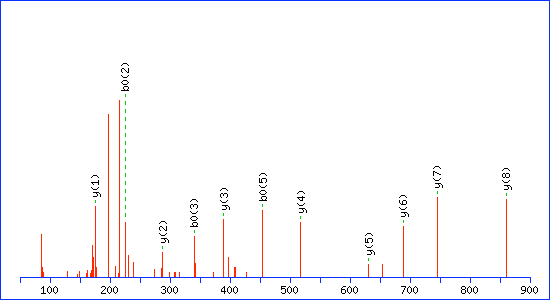


##### Monoisotopic mass of neutral peptide Mr(calc): 1101.5666

**Fixed modifications:** Carbamidomethyl (C)

**Ions Score:** 63 **Expect:** 1.3e-05

**Matches (Bold Red):** 11/86 fragment ions using 15 most intense peaks

| **#** | **b** | **b++** | **b0** | **b0++** | **Seq.** | **y** | **y++** | **y*** | **y*++** | **y0** | **y0++** | **#** |
| --- | --- | --- | --- | --- | --- | --- | --- | --- | --- | --- | --- | --- |
| **1** | 130.0499 | 65.5286 | 112.0393 | 56.5233 | **E** |  |  |  |  |  |  | **10** |
| **2** | 243.1339 | 122.0706 | **225.1234** | 113.0653 | **I** | 973.5313 | 487.2693 | 956.5047 | 478.7560 | 955.5207 | 478.2640 | **9** |
| **3** | 358.1609 | 179.5841 | **340.1503** | 170.5788 | **D** | **860.4472** | 430.7272 | 843.4207 | 422.2140 | 842.4367 | 421.7220 | **8** |
| **4** | 415.1823 | 208.0948 | 397.1718 | 199.0895 | **G** | **745.4203** | 373.2138 | 728.3937 | 364.7005 | 727.4097 | 364.2085 | **7** |
| **5** | 472.2038 | 236.6055 | **454.1932** | 227.6003 | **G** | **688.3988** | 344.7030 | 671.3723 | 336.1898 | 670.3883 | 335.6978 | **6** |
| **6** | 585.2879 | 293.1476 | 567.2773 | 284.1423 | **L** | **631.3774** | 316.1923 | 614.3508 | 307.6790 | 613.3668 | 307.1870 | **5** |
| **7** | 714.3305 | 357.6689 | 696.3199 | 348.6636 | **E** | **518.2933** | 259.6503 | 501.2667 | 251.1370 | 500.2827 | 250.6450 | **4** |
| **8** | 815.3781 | 408.1927 | 797.3676 | 399.1874 | **T** | **389.2507** | 195.1290 | 372.2241 | 186.6157 | 371.2401 | 186.1237 | **3** |
| **9** | 928.4622 | 464.7347 | 910.4516 | 455.7295 | **L** | **288.2030** | 144.6051 | 271.1765 | 136.0919 |  |  | **2** |
| **10** |  |  |  |  | **R** | **175.1190** | 88.0631 | 158.0924 | 79.5498 |  |  | **1** |

MS/MS Fragmentation of SEEEMAAEAAALR

Found in FHIT_HUMAN, Bis(5'-adenosyl)-triphosphatase - Homo sapiens (Human) Match to Query 189: 1376.596438 from(689.305495,2+)


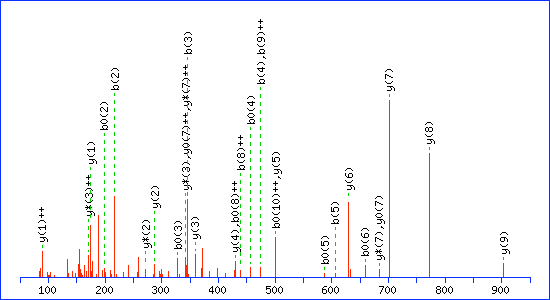


##### Monoisotopic mass of neutral peptide Mr(calc): 1376.6241

**Fixed modifications:** Carbamidomethyl (C)

**Ions Score:** 65 **Expect:** 3.6e-06

**Matches (Bold Red):** 30/110 fragment ions using 34 most intense peaks

| **#** | **b** | **b++** | **b0** | **b0++** | **Seq.** | **y** | **y++** | **y*** | **y*++** | **y0** | **y0++** | **#** |
| --- | --- | --- | --- | --- | --- | --- | --- | --- | --- | --- | --- | --- |
| **1** | 88.0393 | 44.5233 | 70.0287 | 35.5180 | **S** |  |  |  |  |  |  | **13** |
| **2** | **217.0819** | 109.0446 | **199.0713** | 100.0393 | **E** | 1290.5994 | 645.8034 | 1273.5729 | 637.2901 | 1272.5889 | 636.7981 | **12** |
| **3** | **346.1245** | 173.5659 | **328.1139** | 164.5606 | **E** | 1161.5568 | 581.2821 | 1144.5303 | 572.7688 | 1143.5463 | 572.2768 | **11** |
| **4** | **475.1671** | 238.0872 | **457.1565** | 229.0819 | **E** | 1032.5143 | 516.7608 | 1015.4877 | 508.2475 | 1014.5037 | 507.7555 | **10** |
| **5** | **606.2076** | 303.6074 | **588.1970** | 294.6021 | **M** | **903.4717** | 452.2395 | 886.4451 | 443.7262 | 885.4611 | 443.2342 | **9** |
| **6** | 677.2447 | 339.1260 | **659.2341** | 330.1207 | **A** | **772.4312** | 386.7192 | 755.4046 | 378.2060 | 754.4206 | 377.7139 | **8** |
| **7** | 748.2818 | 374.6445 | 730.2712 | 365.6393 | **A** | **701.3941** | 351.2007 | **684.3675** | **342.6874** | **683.3835** | **342.1954** | **7** |
| **8** | 877.3244 | **439.1658** | 859.3138 | **430.1606** | **E** | **630.3570** | 315.6821 | 613.3304 | 307.1688 | 612.3464 | 306.6768 | **6** |
| **9** | 948.3615 | **474.6844** | 930.3509 | 465.6791 | **A** | **501.3144** | 251.1608 | 484.2878 | 242.6475 |  |  | **5** |
| **10** | 1019.3986 | 510.2029 | 1001.3881 | **501.1977** | **A** | **430.2772** | 215.6423 | 413.2507 | 207.1290 |  |  | **4** |
| **11** | 1090.4357 | 545.7215 | 1072.4252 | 536.7162 | **A** | **359.2401** | 180.1237 | **342.2136** | **171.6104** |  |  | **3** |
| **12** | 1203.5198 | 602.2635 | 1185.5092 | 593.2583 | **L** | **288.2030** | 144.6051 | **271.1765** | 136.0919 |  |  | **2** |
| **13** |  |  |  |  | **R** | **175.1190** | **88.0631** | 158.0924 | 79.5498 |  |  | **1** |

MS/MS Fragmentation of **FGVAPDHPEVK**

Found in **ADRO_HUMAN**, NADPH:adrenodoxin oxidoreductase, mitochondrial precursor - Homo sapiens (Human)

Match to Query 144: 1194.560128 from(598.287340,2+)


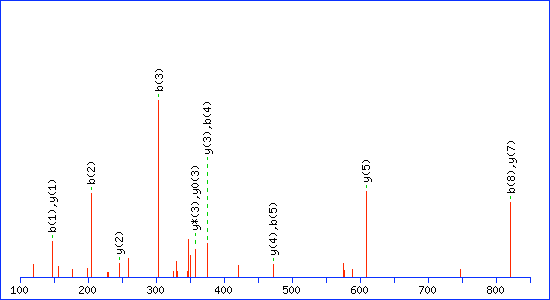


##### Monoisotopic mass of neutral peptide Mr(calc): 1194.6033

**Fixed modifications:** Carbamidomethyl (C)

**Ions Score:** 47 **Expect:** 0.00049

**Matches (Bold Red):** 14/86 fragment ions using 20 most intense peaks

| **#** | **b** | **b++** | **b0** | **b0++** | **Seq.** | **y** | **y++** | **y*** | **y*++** | **y0** | **y0++** | **#** |
| --- | --- | --- | --- | --- | --- | --- | --- | --- | --- | --- | --- | --- |
| **1** | **148.0757** | 74.5415 |  |  | **F** |  |  |  |  |  |  | **11** |
| **2** | **205.0972** | 103.0522 |  |  | **G** | 1048.5422 | 524.7747 | 1031.5156 | 516.2615 | 1030.5316 | 515.7694 | **10** |
| **3** | **304.1656** | 152.5864 |  |  | **V** | 991.5207 | 496.2640 | 974.4942 | 487.7507 | 973.5102 | 487.2587 | **9** |
| **4** | **375.2027** | 188.1050 |  |  | **A** | 892.4523 | 446.7298 | 875.4258 | 438.2165 | 874.4417 | 437.7245 | **8** |
| **5** | **472.2554** | 236.6314 |  |  | **P** | **821.4152** | 411.2112 | 804.3886 | 402.6980 | 803.4046 | 402.2060 | **7** |
| **6** | 587.2824 | 294.1448 | 569.2718 | 285.1396 | **D** | 724.3624 | 362.6849 | 707.3359 | 354.1716 | 706.3519 | 353.6796 | **6** |
| **7** | 724.3413 | 362.6743 | 706.3307 | 353.6690 | **H** | **609.3355** | 305.1714 | 592.3089 | 296.6581 | 591.3249 | 296.1661 | **5** |
| **8** | **821.3941** | 411.2007 | 803.3835 | 402.1954 | **P** | **472.2766** | 236.6419 | 455.2500 | 228.1287 | 454.2660 | 227.6366 | **4** |
| **9** | 950.4367 | 475.7220 | 932.4261 | 466.7167 | **E** | **375.2238** | 188.1155 | **358.1973** | 179.6023 | **357.2132** | 179.1103 | **3** |
| **10** | 1049.5051 | 525.2562 | 1031.4945 | 516.2509 | **V** | **246.1812** | 123.5942 | 229.1547 | 115.0810 |  |  | **2** |
| **11** |  |  |  |  | **K** | **147.1128** | 74.0600 | 130.0863 | 65.5468 |  |  | **1** |


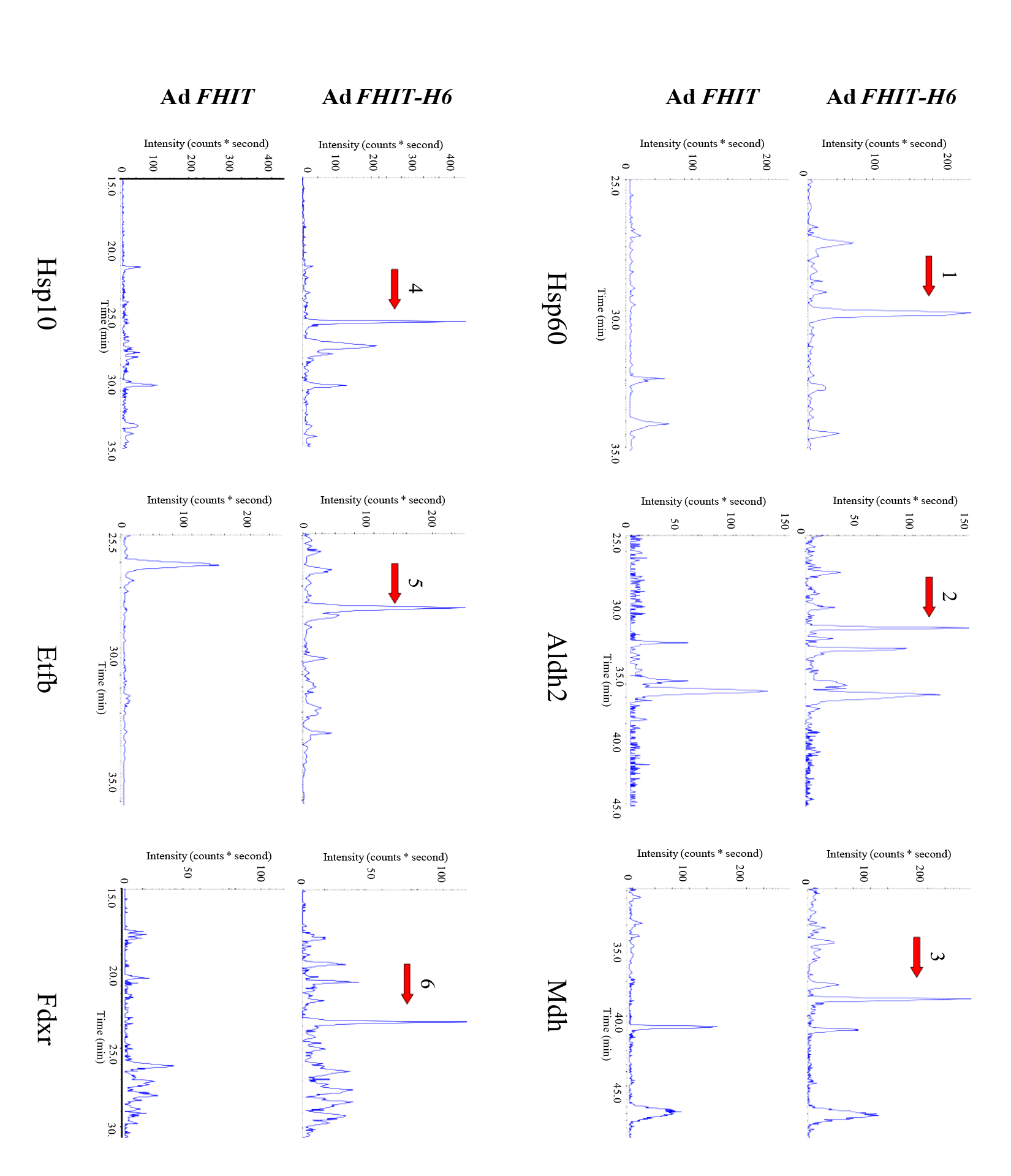
Initial validation of candidate Fhit protein partners identified through nanobore LC- MS/MS. Selected ion chromatograms (SIC) for AdFHIT-His6 and control samples are shown. The six SICs pairs report ion currents of the six following m/z values: 1) 672.8 (peak at retention time 30 min. was identified as tryptic peptide TVIIEQSWGSPK belonging to Hsp60), 2) 685.4 (peak at retention time 32 min. identified as tryptic peptide LGPALATGNVVVMK belonging to Aldh2), 3) 617.3 (peak at retention time 39 min. identified as tryptic peptide IFGVTTLDIVR belonging to Mdh), 4) 658.4 (peak at retention time 26 min. identified as tryptic peptide VLQATVVAVGSGSK belonging to Hsp10), 5) 551.7 (peak at retention time 28 min. identified as tryptic peptide EIDGGLETLR belonging to Etfb), 6) 598.3 (peak at retention time 23 min. identified as tryptic peptide FGVAPDHPEVK belonging to Fdxr). For more information on the MS/MS identification of the selected peaks, see MS/MS spectra in Supplementary Information, Methods. Peptides of interest, indicated by red arrows, are exclusively present in AdFHIT-His6 sample.
